# Complete Genome Sequence of *Mycobacterium avium* subsp. *paratuberculosis*, Isolated from Human Breast Milk

**DOI:** 10.1128/genomeA.01252-13

**Published:** 2014-02-06

**Authors:** John P. Bannantine, Lingling Li, Michael Mwangi, Rebecca Cote, Juan A. Raygoza Garay, Vivek Kapur

**Affiliations:** a National Animal Disease Center-USDA-ARS, Ames, Iowa, USA; b Department of Veterinary and Biomedical Science, Pennsylvania State University, University Park, Pennsylvania, USA

## Abstract

*Mycobacterium avium* subsp. *paratuberculosis* is the etiologic agent of Johne’s disease in ruminants and has also been associated with human Crohn’s disease. We report the complete genome sequence of *M. avium* subsp. *paratuberculosis*, isolated from the breast milk of a Crohn’s disease patient. This sequence has high identity with characterized strains recovered from cattle.

## GENOME ANNOUNCEMENT

*Mycobacterium avium* subsp. *paratuberculosis* causes Johne’s disease (JD) in cattle, sheep, goats, and other ruminant animals. JD presents as a chronic granulomatous intestinal infection with a worldwide distribution and imposes a significant economic toll on livestock industries (1). *M. avium* subsp. *paratuberculosis* has a complex cell wall structure containing mycolic acids and several lipids similar to those of other members of this genus, yet it is the most slowly growing member. This bacterium often requires 8 to 16 weeks before colonies are visible in culture, which is a major hurdle in diagnostics and therefore in the implementation of optimal JD control measures. Although a well-established domestic and wild animal pathogen, it has also been implicated as a causative agent in human Crohn’s disease (2), and even though this link is controversial (3), *M. avium* subsp. *paratuberculosis* isolates have been obtained from humans. For instance, *M. avium* subsp. *paratuberculosis* 4, the isolate whose sequence we report here, was originally isolated from the breast milk of a Crohn’s disease patient in 2000 (4).

*M. avium* subsp. *paratuberculosis* is a member of the *M. avium* complex (5), yet only the subspecies *paratuberculosis* of this complex has been found to cause Johne’s disease. Another distinguishing phenotype of *M. avium* subsp. *paratuberculosis* is its requirement for the siderophore mycobactin in laboratory medium for growth. The genomes of cattle, sheep, and human strains of this pathogen have been sequenced (6–9). However, the human strain sequences are only in draft form. The data suggest that all nonsheep isolates of *M. avium* subsp. *paratuberculosis* are highly conserved and thus cluster as a homogeneous group (10), and isolates from humans are thought to cluster with the bovine strains; however, a complete sequence of an isolate from humans has not been available, and this has precluded comprehensive and definitive analyses.

Purified genomic DNA obtained from strain *M. avium* subsp. *paratuberculosis* 4 was subjected to whole-genome shotgun sequencing using 454 Life Sciences GS20 pyrosequencing technology (Roche, Indianapolis, IN). The 88.5 million bp were assembled into ~400 contigs using Newbler assembly software (Roche). These contigs were assembled into ~50 scaffolds using the *M. avium* subsp. *paratuberculosis* strain K-10 sequence as a reference. Sanger-based sequencing was used to close all gaps in an iterative manner, and areas with low-quality scores were resequenced in order to obtain a single assembled high-quality genome sequence that totals 4.83 Mb. The gaps in each scaffold were first closed by using the Lasergene SeqMan software 9.0 (DNAstar, Madison, WI). The gaps between scaffolds were closed by PCR amplification of the entire insertion sequence (IS) elements or repetitive sequences and flanking region on each end of the scaffolds. An annotation file (Artemis) was generated by Rapid Annotations using Subsystems Technology (http://rast.nmpdr.org/rast.cgi), and each coding sequence (CDS) was manually verified (11).

### Nucleotide sequence accession number.

The whole-genome sequence of *M. avium* subsp. *paratuberculosis* 4 has been deposited at DDBJ/EMBL/GenBank under the accession no. CP005928.1.
